# Supplementary figures and images for: Genomic, epidemiological and digital surveillance of Chikungunya virus in the Brazilian Amazon
Source: PLoS Negl Trop Dis. 2019 Mar 7;13(3):e0007065. doi: 10.1371/journal.pntd.0007065 (PMC6424459; doi:10.1371/journal.pntd.0007065)

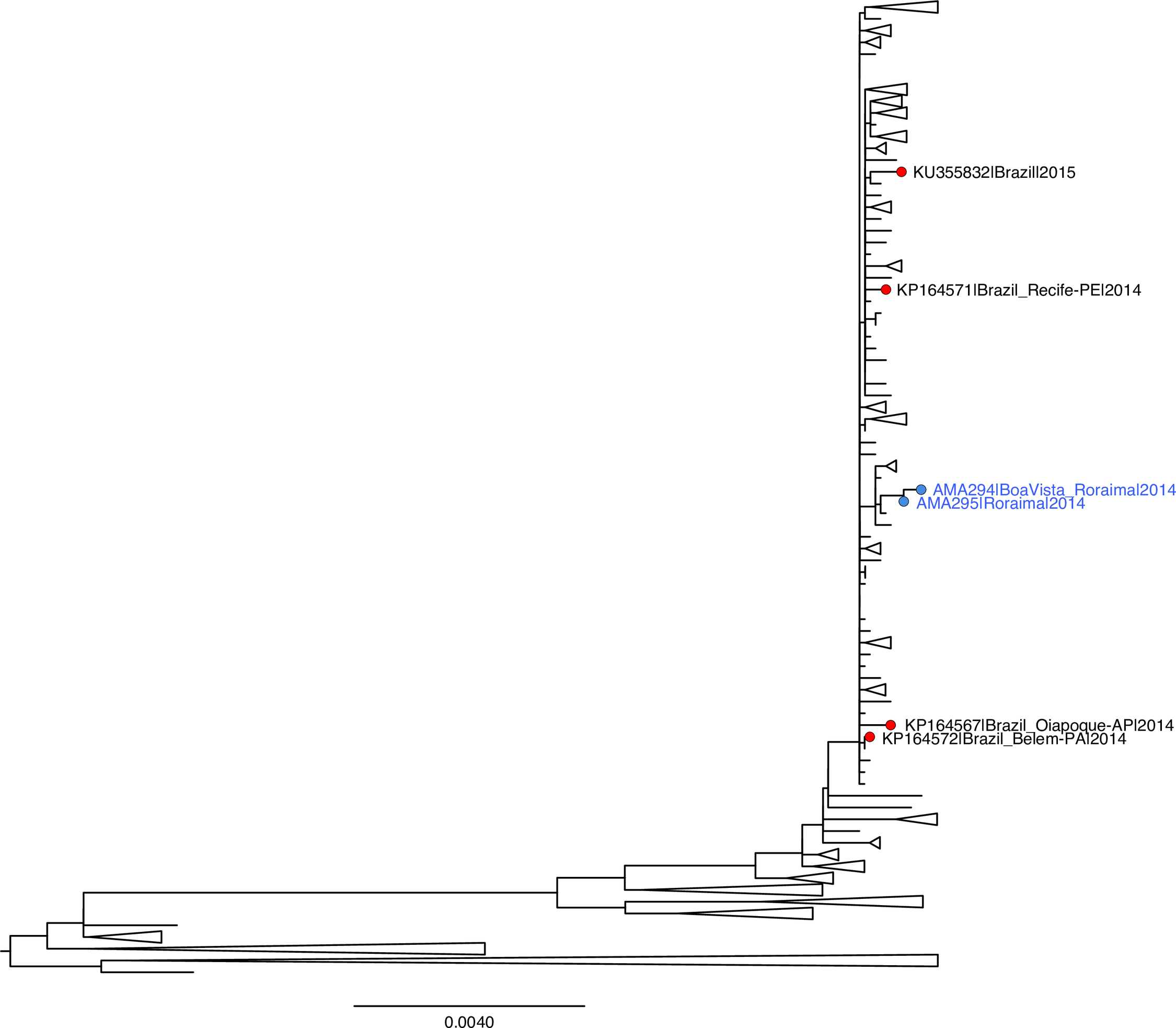

Supplement: S1 Fig — Includes isolates from Southeast Asia, Americas and Brazil. Isolates represented by blue tips were sampled in Roraima, while isolates shown in red represent other strains sampled in Brazil. (TIF) [file pntd.0007065.s001.tif]

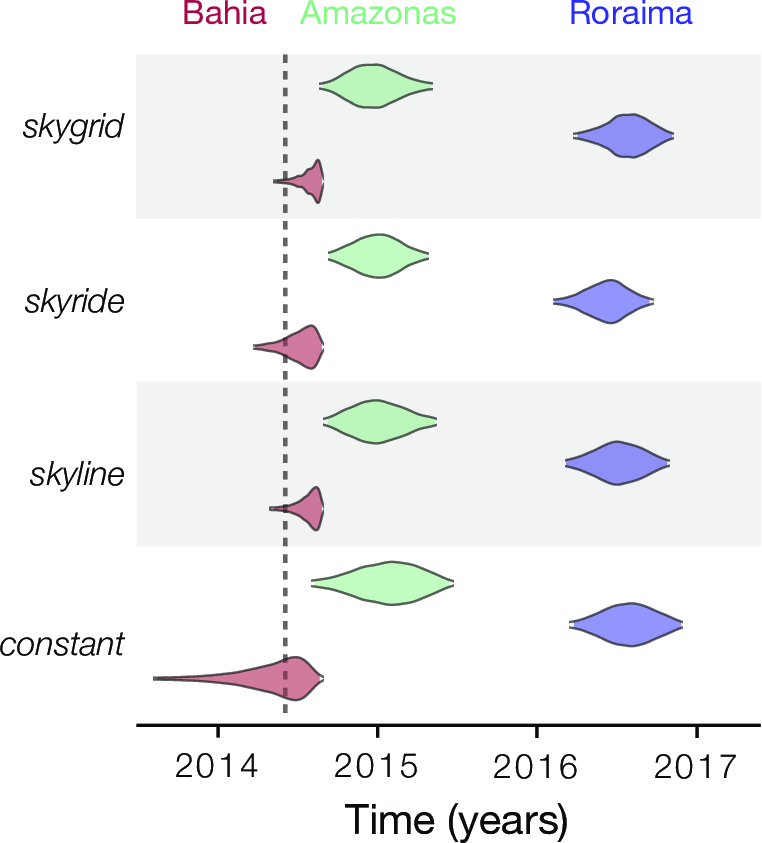

Supplement: S2 Fig — Estimates for node A (time of the most recent common ancestor, in dark red, see Fig 3B), node B (main Amazonas clade, in green), and node C (Roraima clade, in purple) are shown for different non-parametric models (Bayesian skygrid, skyride, skyline) and for a simple constant population size model. (TIF) [file pntd.0007065.s002.tif]

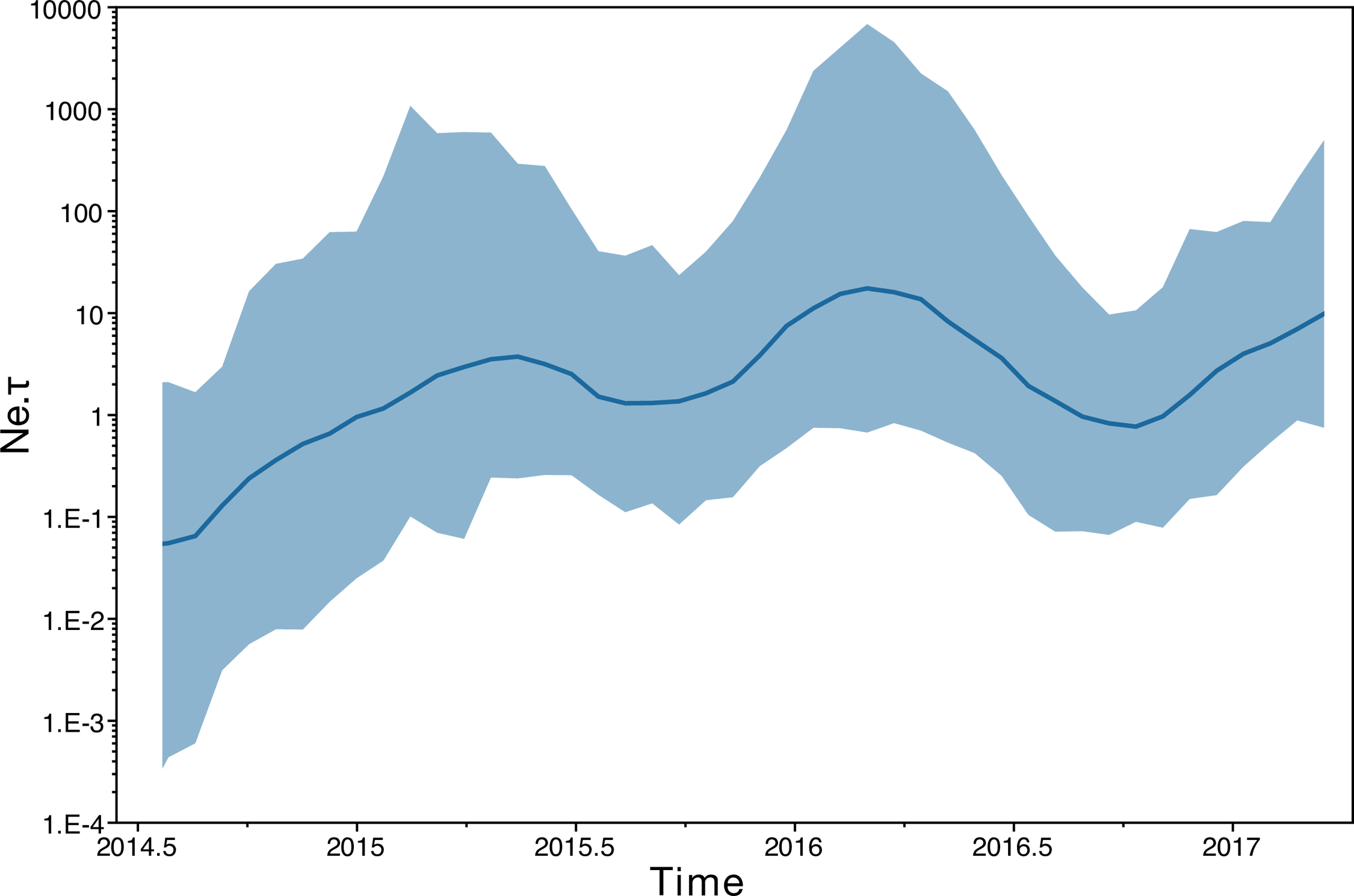

Supplement: S3 Fig — Fluctuation of effective population size over time as inferred through a Bayesian skygrid coalescent model. (TIF) [file pntd.0007065.s003.tif]

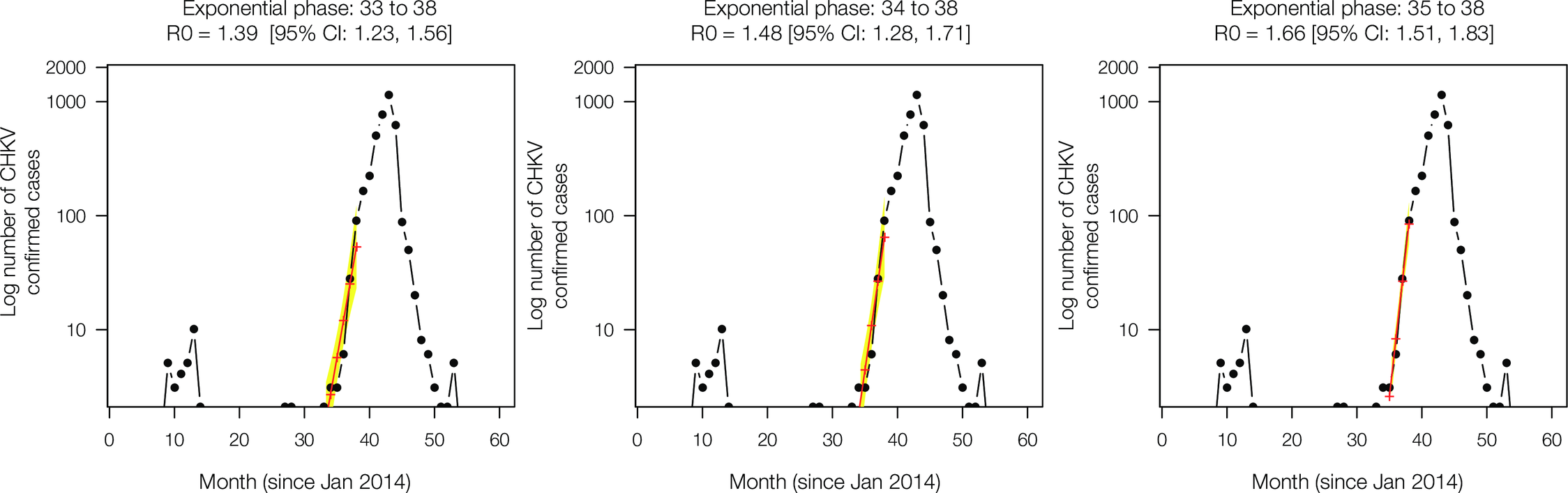

Supplement: S4 Fig — Log number of notified cases per month are plotted against number of months since January 2015. (TIF) [file pntd.0007065.s004.tif]

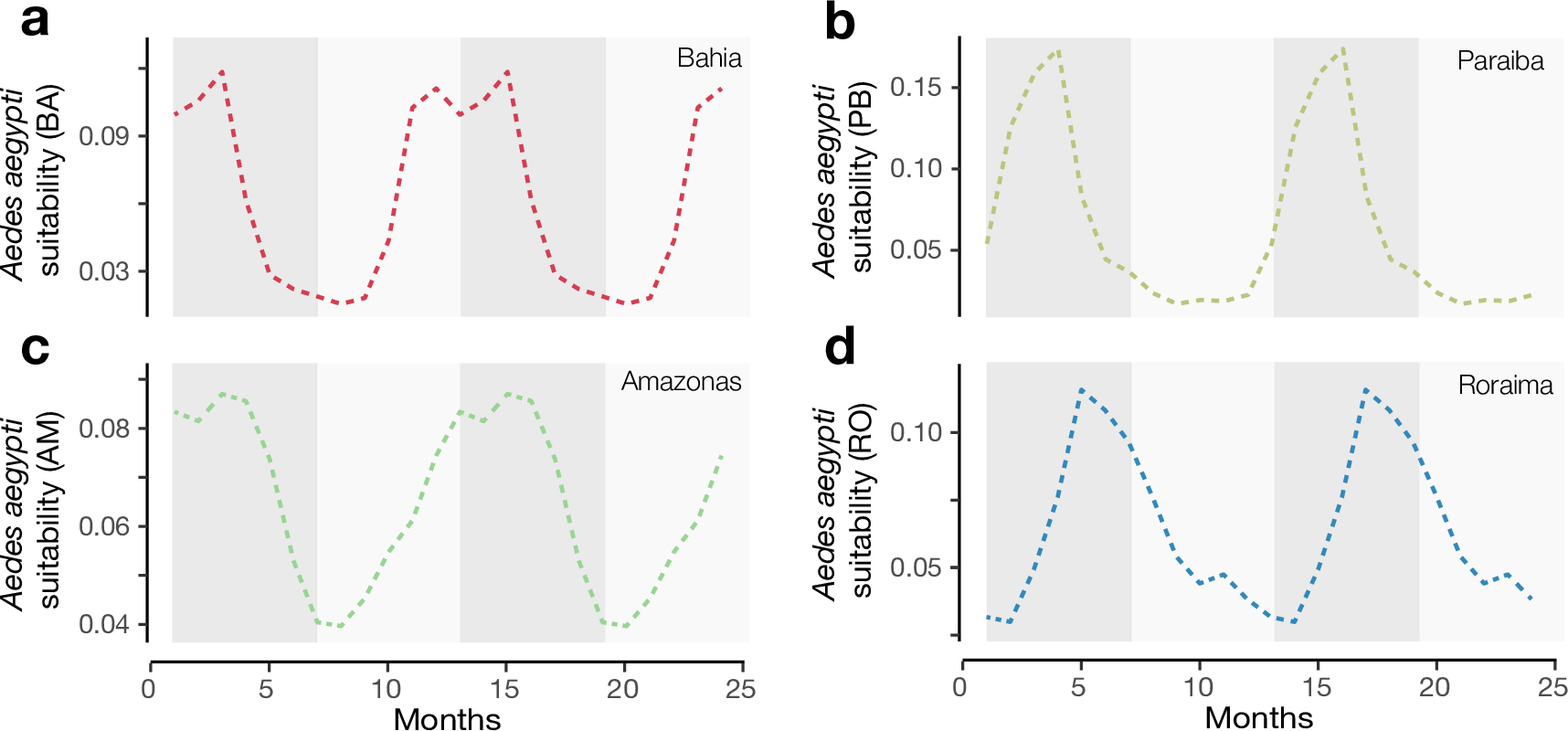

Supplement: S5 Fig — (TIF) [file pntd.0007065.s005.tif]
