# Supplementary material for: Genomic, epidemiological and digital surveillance of Chikungunya virus in the Brazilian Amazon
Source: PLoS Negl Trop Dis. 2019 Mar 7;13(3):e0007065. doi: 10.1371/journal.pntd.0007065 (PMC6424459; doi:10.1371/journal.pntd.0007065)
Supplement: S1 Table — (DOCX) [file pntd.0007065.s006.docx]

**Table S1. Minion sequencing statistics.**

| Isolate | Mapped reads | Average depth coverage | Bases covered >10x | Bases covered > 25x | Reference covered (%) |
| --- | --- | --- | --- | --- | --- |
| AMA290 | 13624 | 767 | 10276 | 10258 | 90.2 |
| AMA291 | 60261 | 2047 | 9489 | 9280 | 80.7 |
| AMA292 | 68090 | 2746 | 10402 | 10223 | 90.2 |
| AMA293 | 64953 | 2096 | 9745 | 9701 | 84.4 |
| AMA294 | 21361 | 701 | 10252 | 10022 | 90.2 |
| AMA295 | 16370 | 531 | 10188 | 10077 | 90.2 |
| AMA74 | 42276 | 1951 | 10396 | 10195 | 90.2 |
| AMA346 | 31210 | 1225 | 10243 | 10208 | 90.2 |
| AMA350 | 63672 | 1673 | 7522 | 7168 | 54.7 |
| AMA352 | 13530 | 536 | 10219 | 10184 | 88.6 |
| AMA354 | 22214 | 752 | 10082 | 9985 | 86.9 |
| AMA362 | 9938 | 398 | 10237 | 10128 | 88.6 |
| AMA364 | 28494 | 1079 | 9813 | 9577 | 83.3 |
| AMA366 | 38228 | 1441 | 10264 | 10224 | 90.0 |
| AMA368 | 12968 | 503 | 11122 | 10825 | 93.1 |
| AMA369 | 7280 | 311 | 11225 | 11149 | 95.6 |
| AMA374 | 7030 | 305 | 10225 | 8805 | 71.4 |
| AMA379 | 7970 | 348 | 11226 | 11092 | 96.1 |
| AMA381 | 7522 | 327 | 11214 | 11208 | 97.7 |
| AMA382 | 14040 | 411 | 9915 | 9424 | 76.6 |
